# Medication non-adherence and associated factors among older adult stroke survivors in China

**DOI:** 10.3389/fphar.2022.1054603

**Published:** 2022-11-24

**Authors:** Wenjing Cao, Azidah Abdul Kadir, Juan Wang, Lin Hu, Linlan Wen, Mei Yu, Liqun Peng, Lanying Chen, Na Luo, Intan Idiana Hassan

**Affiliations:** ^1^ School of Health Sciences, Health Campus, Universiti Sains Malaysia, Kelantan, Malaysia; ^2^ Xiang Nan University, Chenzhou, Hunan, China; ^3^ School of Medical Sciences, Health Campus, Universiti Sains Malaysia, Kelantan, Malaysia; ^4^ Guangdong Pharmaceutical University, Guangzhou, Guangdong, China; ^5^ Chenzhou No.1 People’s Hospital, Chenzhou, Hunan, China; ^6^ Chenzhou Third People’s Hospital, Chenzhou, Hunan, China; ^7^ Affiliated hospital of Xiangnan University, Chenzhou, Hunan, China

**Keywords:** medication, non-adherence, older adults, stroke survivors, associated factors

## Abstract

**Aim:** Medication non-adherence has remained a common and costly global health issue of growing importance among older adults. This study aims to determine the prevalence and associated factors related to medication non-adherence among older adult stroke survivors in China.

**Methods and results:** In this cross-sectional study, a total of 402 older adult stroke survivors were recruited from three tertiary hospitals in China. The results of the survey showed that 61.4% exhibited medication non-adherence. The chances of medication non-adherence among older adult stroke survivors who had primary school or less educational levels were higher than those who had senior secondary and junior college educational levels [OR (95% CI) = 0.440(0.249, 0.778)] as well as those who had a bachelor’s degree or above educational levels [OR (95%CI) = 0.367(0.202, 0.667)]. Moreover, the probability of medication non-adherence with 4–5 and ≥6 types of total prescription medications per day increased by 1.993 times [OR (95% CI) = 1.993(1.190, 3.339))] and 2.233 times [OR (95%CI) = 2.233(1.159, 4.300)], respectively, as compared to when there were ≤3 types. Furthermore, medication non-adherence decreased with the increase in health literacy scores (*β* = −0.641 (95% CI; (0.913, 0.965)) and BMQ specific-necessity scores (*β* = −0.131 (95% CI; 0.806, 0.995)). On the other hand, when the BMQ specific-concerns score increased by one unit, medication non-adherence increased by 11.1% [OR (95% CI) = 1.111(1.044, 1.182)].

**Conclusion:** The present study found that patient medication adherence among older adult stroke survivors in China is problematic and associated with educational levels, total prescribed drugs per day, beliefs about medication, and health literacy scores. This indicates that measures should be taken to enhance medication adherence among such higher-risk populations.

## Introduction

Globally, stroke remains the second leading cause of death and the third leading cause of disability in adults (Zhang et al., 2021; Feigin et al., 2022). In 2019, stroke was responsible for 143 million disability-adjusted life-years and 6·55 millions of deaths (Feigin et al., 2022). In this context, many published studies have concluded that multiple modifiable or non-modifiable factors may increase the risk of stroke occurrences. Here, it is worth mentioning that approximately three-quarters of all strokes occur in persons older than 65 years (Yousufuddin and Young, 2019). Additionally, Gorelick (2019) suggested that to relieve the future global burden of strokes, prevention among older adults would be an important objective.

Stroke survivors are at an elevated risk of having recurrences (Del et al., 2019), which make up 25–30% of all strokes (Hankey, 2014). As recurrent strokes are associated with high mortality and more disabling (Wu et al., 2019), prescribed medications are recommended for secondary prevention of stroke by guidelines (Coutts et al., 2015; Ahmed et al., 2019). Moreover, several sources of evidence indicate that secondary prevention medications tend to reduce the risk of stroke recurrence (Bushnell et al., 2014; Flach et al., 2020; Yeo et al., 2020). For example, it has been conclusively shown that higher adherence to antithrombotic or statin treatments is associated with a decreased risk of stroke recurrence and mortality (Yeo et al., 2020; Rodriguez-Bernal et al., 2021). In the same vein, Toyoda et al. (2019) reported that the combination of cilostazol with aspirin or clopidogrel resulted in a reduced recurrence of ischemic strokes. In addition, a review conducted by Katsanos and Hart (2020) indicated that an important protection against recurrent strokes is provided by the current triad of pharmacologic mainstays for secondary stroke prevention: blood-pressure lowering drugs, statin drugs, and antiplatelet agents. Meanwhile, there is consensus among scientists that non-medication adherence is a potential modifiable risk factor for poor BP control (Boima et al., 2015; Burnier and Egan, 2019), which is itself a well-established and modifiable risk factor for strokes.

However, patient adherence to medication is largely accounted for the effect of medical management to prevent recurrent strokes and other adverse outcomes (Zhang et al., 2021). Medication adherence is generally defined as the extent to which patients take their medication in line with the recommendations of their healthcare provider (Sabaté, 2003). A large and growing body of literature has reported that post-stroke medication adherence and persistence rates are low in stroke survivors (Kim et al., 2020; Ruksakulpiwat et al., 2020; Zhang et al., 2021). These results, therefore, suggest that improving medication adherence among survivors of stroke should be a growing concern to clinicians, healthcare systems, and other stakeholders (e.g., payers).

There is no doubt that a key step in the creation of an appropriate strategy to improve medication adherence is first understanding stroke survivors’ non-medication adherence and its associated factors. Although several studies have shown the prevalence and factors associated with medication adherence among stroke survivors (Pan et al., 2017; Wei et al., 2017; Ruksakulpiwat et al., 2020), they are not specific to older adults. Furthermore, it is important to note that medication adherence is of a particular concern in older persons. A recent systematic review indicated factors negatively associated with adherence in this population: complex regimens with multiple prescribing physicians; problems with medication storage and formulation; and multimorbidity and cognitive impairment (Smaje et al., 2018). On the other hand, non-medication adherence rates among older adults in other chronic diseases (e.g., diabetes mellitus and hypertension patients) have been found to be alarmingly high (Saqlain et al., 2019; Xu et al., 2020). Meanwhile, older adult stroke survivors are likelier to have other chronic medical conditions (e.g., hypertension, history of cardiac-related comorbidities, diabetes, etc.) (Gruneir et al., 2016; Maresova et al., 2019), which means they have to take more medications to meet their broader health needs, thus further challenging their adherence to medications and an increase in the possibilities of worse health-related risks when non-adherence occurs. However, little is known about the prevalence of non-medication adherence and the factors associated with it among older adult stroke survivors, which this study aims to examine among older adult stroke survivors in Chenzou, Hunan, China.

## Methods

### Study design

We conducted a cross-sectional study from June 2022 to August 2022. The study was approved by the ethics committee of three tertiary hospitals (Affiliated Hospital of Xiangnan University, Chenzhou No. 1 People’s Hospital, and Chenzhou Third People’s Hospital) before the initiation of this study.

### Participants

Participants were recruited using a systematic sampling method from three tertiary hospitals in Chenzhou, Hunan Province, China. They were eligible for inclusion based on the following criteria: if they were aged 60 years or older; had a history of strokes confirmed by neuroimaging at the time of the episode; taken at least one medication in the previous month such as (but not limited to) anti-platelets, statins, and anti-hypertensives to control risk factors for strokes; it had been more than a month since the last stroke episode; were able to read Chinese and communicate in Mandarin Chinese or the local Chenzhou dialect. We excluded the following patients who: had psychiatric illness or deafness, aphasia, or other language barriers; had cognitive impairment (Mini-Mental State Examination score ≤17 [for illiterate] or ≤20 [individuals with 1–6 years of education] or ≤24 [individuals with 7 or more years of education]).

### Sample size calculation

Sample size calculations were conducted to determine the prevalence of medication non-adherence using a single proportion formula and the associated factors using PS Power and Sample Size software version 3.1.6 for a dichotomous two-proportion formula. Based on the calculations, the highest sample size of 416 was chosen. This sample size was based on a precision of 0.05 and power of 80%; the proportion of permanent employment being 0.448 was based on the study by Kim et al. (2020). However, after considering the non-response level of 10%, the calculated sample size is 458.

### Survey instrument

The study protocol included one set of demographic questions and three validated instruments—the General Medication Adherence Scale (GMAS-C), Beliefs about Medicines Questionnaire (BMQ), and Health Literacy Scale for Stroke Patients.

Demographic data were self-reported by the participants and included gender, age, marital status, educational level, duration of disease, living conditions, membership in ethnic groups, payment method for medical expenses, per-capita monthly household income, occupation status, number of prescribed medicines, frequency of daily doses, types of strokes, residence, and existence of comorbidities.

The GMAS-C is a self-report tool containing 11 items that measure medication adherence among patients with chronic diseases, including strokes. It consists of three dimensions: 1) patient behavior-related non-adherence (five items), 2) additional disease and pill burden (four items), and 3) cost-related non-adherence (two items). All items are answered on a four-point Likert scale: responses of “always,” “mostly,” “sometimes,” and “never” are scored as 0, 1, 2, and 3, respectively. The total GMAS-C score is the summation of the scores for the 11 items and ranges from 0 to 33: high adherence (30–33), good adherence (27–29), partial adherence (17–26), low adherence (11–16), and poor adherence (0–10). Patients with a total score of 26 and below are considered medication non-adherent, while a score of 27 and above indicates adherence (Naqvi et al., 2019).

Moreover, Wang et al. (2021) undertook validity and reliability studies of the Chinese version of the GMAS-C in 2021 and found that the exploratory factor analysis extracted three factors with eigenvalues >1 and that 60% of the total variance was explained by a three-factor solution. Next, confirmatory factor analysis showed acceptable fit indices (χ^2^/df = 1.58, IFI = 0.96, TLI = 0.94, CFI = 0.96, and RMSEA = 0.05). Thus, it was concluded that the scale was a valid and reliable instrument for the assessment of medication adherence.

The BMQ was developed to assess personal beliefs and worries about taking medications for diseases (Horne and Weinman, 1999a). It consists of two domains—the BMQ-Specific and BMQ General—that are independently validated and can be used in combination or separately. In this study, we used only the BMQ-Specific because Horne and Weinman (1999a) reported that it is a flexible instrument that can be adapted to assess beliefs about all medicines for a particular condition or individual components of a regimen. The Chinese version of the BMQ-Specific has been proved to be a good instrument with acceptable reliability and validity. The Cronbach’s α coefficients of the necessary and concerns dimensions were 0.813 and 0.706, respectively, and the test–retest reliability coefficients were 0.743 and 0.786, respectively (Yang, et al., 2014). The BMQ-Specific has two subscales (specific-necessity and the specific-concerns) with five questions each, which aim to assess beliefs about the necessity of prescribed medication and concerns about them based on beliefs about the danger of dependence, long-term toxicity, and the disruptive effects of medication (Verhagen, 2018).

The Chinese version of the Health Literacy Scale for Stroke Patients has been used to assess the health literacy of patients who have had a stroke and proved to be a good instrument with acceptable reliability and validity (Jiru, et al., 2020). It has three subscales and a total of 20 items. The first subscale is basic knowledge of strokes, which consists of six items. A five-point Likert scale is used, and responses are assigned to a score of 5 for “strongly agree,” 4 for “agree,” 3 for “neutral,” 2 for “disagree,” and 1 for “strongly disagree.” For two items (4,5), reverse scoring is applied. The second subscale with nine items evaluates the healthy lifestyles and behaviors of stroke survivors: it is responded to as follows: 1—“never”; 2—“rarely”; 3—“sometimes”; 4—“often”; and 5—“always.” Reverse coding was carried out for item 2 because it is a negative statement. The third subscale is related to the basic skills of stroke survivors and consists of five items, for which a five-point Likert score method is used. The response options are “never,” “rarely,” “sometimes,” “often,” and “always,” which corresponds to the scores of 1, 2, 3, 4, and 5 points, respectively. A higher score indicates better health literacy.

### Data collection procedure

Data were collected using a questionnaire-guided interview method. Written informed consent was received prior to the respondents answering the questionnaire. This study was conducted in full accordance with the principles of the Declaration of Helsinki. The participants were informed that their participation was voluntary and that they had the right to withdraw from the study at any time without any influence, coercion, or persuasion. Questionnaires were distributed and completed by participants (it took approximately 20 min to complete the questionnaires) who were in rehabilitation settings, at the departments of neurology, or at the out-patient clinics during the investigation period. A total of 458 stroke survivors who were admitted to hospitals were approached. Five trained health professionals who speak the local language and did not work at the study hospital were recruited to distribute and collect questionnaires. In order to familiarize data collectors the data collection tool, we conducted training prior to the study. Regarding illiterate participants or those with eye problems, the question items were read word by word exactly as they appeared on the questionnaires by the researchers. Responses were then recorded on the questionnaire. Upon completion, the questionnaires were collected immediately and checked for any missing information, after which follow-ups were undertaken with the participants if needed.

### Statistical analysis

All statistical analyses were performed using the Statistical Program for the Social Sciences (SPSS version 26). The data were checked, explored, and cleaned. Descriptive statistics was used to calculate the frequency and percentage (categorical data) or the mean and standard deviations (continuous data). The prevalence of medication adherence was calculated using the aforementioned cutoff scores and reported as the percentage of cases in different populations. The 95% CIs were produced using exact binomial methods. The chi-squared test method was used for univariate analysis. To explore factors that are potentially associated with medication non-adherence, binary logistic regression analyses were performed, and odds ratios (ORs) and 95% CIs were presented. *p*-values less than 0.05 were considered statistically significant throughout the analysis.

## Results

### Characteristics of participants

A total of 458 older adult stroke survivors were approached, with 402 giving consent and being enrolled in the study (response rate of 87.8%). Most participants were male (243 [60.4%]), were married (293 [72.9%]), were of Han nationality (350 [87.5%]), and had comorbidities (334 [83.1%]). Of the 402 responding participants, 341 (84.8%) had ischemic strokes, while 61 (15.2%) had hemorrhagic strokes. Meanwhile, 43.5% resided in city areas, and 42.0% took medicine only one time a day. Almost half the participants (211 [52.5%]) were unemployed. Approximately one-third (39.2%) only had a primary school education or less. Those living with spouses represented 51.0% of the sample, and 43.5% used rural cooperative medical insurance. A total of 184 participants (45.8%) had a monthly per-capita income between 1,001 and 3,000 ¥. The mean age of participants was 72.16 ± 7.43 SD, the mean (SD) duration of stroke disease was 49.37 (51.99) months, and the mean total types of prescription medications per day was 3.96 (1.68). The characteristics of the study participants are shown in Table 1.

**TABLE 1:** Demographic characteristics of total participants (*N* = 402).

Variable	*n*	(%) OR mean ± SD
Age, y		72.16 ± 7.43
Gender		
Male	243	60.4
Female	159	39.6
Marital status		
Unmarried	1	0.2
Married	293	72.9
Divorced or widowed	108	26.9
Educational level (missing = 1)		
Primary school or less	157	39.2
Junior school	121	30.2
Senior secondary and junior college	97	24.2
Bachelor degree or above	26	6.5
Duration of disease, month		49.37 ± 51.99
Living conditions		
Living alone	73	18.2
Living with spouse	205	51.0
Living with children	48	11.9
Living with a nanny	1	0.2
Living with spouse and children	75	18.7
Ethnic groups (missing = 2)		
Han nationality	350	87.5
Others	50	12.5
Payment methods		
Self-pay	2	0.5
Urban resident basic medical insurance	81	20.1
Rural cooperative medical insurance	175	43.5
Urban employee basic medical insurance	141	35.1
Public medical care	3	0.7
Monthly household income per capita, ¥^a^		
≤1,000	90	22.4
1,001–3,000	184	45.8
3,001–4999	103	25.6
≥5000	25	6.2
Occupation status		
Employed	29	7.2
Unemployed	211	52.5
Retired	162	40.3
Residence (missing = 1)		
Rural areas	107	26.7
Town and county	119	29.7
City	175	43.5
Comorbidity		
No	68	16.9
Yes	334	83.1
Total prescription medications per day		3.96 ±1.68
Frequency of daily doses		
1	169	42.0
2	96	23.9
3	129	23.9
4	8	2.0
Ischemic stroke	341	84.8
Hemorrhagic stroke	61	15.2

^a^ As of 30 August 2022, 1¥ = 0.145US$.

### Participants’ medication adherence

Of the 402 responding participants, 247 (61.4%) exhibited medication non-adherence. In total, 22.6% of the participants had high adherence, approximately 64 (15.9%) of the participants had good adherence, and 149 (37.1%) had partial adherence. Meanwhile, low adherence and poor adherence were 13.7% and 10.7%, respectively. The participants’ medication adherence is detailed in Table 2.

**TABLE 2:** Medication adherence among patients.

GMAS score	Mean ± SD	High adherence no. (%)	Good adherence no. (%)	Partial adherence no. (%)	Low adherence no. (%)	Poor adherence no. (%)	Non-adherence no. (%)	Adherence no. (%)
Patient behaviour related non-adherence	15.00 ± 9.83	129(32.1)	82(20.4)	80(19.9)	55(13.7)	56(13.9)	–	–
Additional disease and pill burden	12.00 ± 8.19	109(27.1)	100(24.9)	115(28.6)	38(9.5)	40(10)	–	–
Cost-related non-adherence	6.00 ± 4.60	179(44.5)	40(10.0)	129(32.1)	37(9.2)	17(4.2)	–	–
Overall adherence	22.62 ± 7.77	91(22.6)	64(15.9)	149(37.1)	55(13.7)	43(10.7)	247(61.4)	155(38.6)

### Relationship of non-adherence with related factors

Table 3 shows the univariate analysis of the factors associated with medication non-adherence, which are as follows: educational level (*p* < 0.001), payment methods (*p* < 0.001), total prescription medications per day (*p* = 0.002), monthly income per capita (*p* < 0.001), occupation status (*p* < 0.001), residence (*p* < 0.001), BMQ specific-necessity score (*p <* 0.001), BMQ specific-concerns score (*p* = 0.002), and health literacy score (*p* < 0.001).

**TABLE 3:** Relationship of non-adherence with related factors.

Variable	Non-adherence	Adherence	Total, no. (%)	χ^2^	*p*
Age				0.781	0.677
≤70	108 (60.7)	70 (39.3)	178 (44.3)		
71∼79	99 (63.9)	56 (36.1)	155 (38.6)		
≥80	40 (58.0)	29 (42.0)	69 (17.2)		
Gender				0.004	0.949
Male	149 (61.3)	94 (38.7)	243 (60.4)		
Female	98 (61.6)	61 (38.4)	159 (39.6)		
Marital status				0.487	0.485
Married	177 (60.4)	116 (39.6)	293 (72.9)		
Unmarried, divorced, or widowed	70 (64.2)	39 (35.8)	109 (27.1)		
Educational level				29.926	<0.001
Primary school or less	121 (77.1)	36 (22.9)	157 (39.2)		
Junior school	69 (57)	52 (43)	121 (30.2)		
Senior Secondary and junior college	47 (48.5)	50 (51.5)	97 (24.2)		
Bachelor degree or above	10 (38.5)	16 (61.5)	26 (6.5)		
Duration of disease, month				4.370	0.112
≤12	74 (55.2)	60 (44.8)	134 (33.3)		
13∼36	79 (68.1)	37 (31.9)	116 (28.9)		
≥37	94 (61.8)	58 (38.2)	152 (37.8)		
Living conditions				0.002	0.969
Living alone	45 (61.6)	28 (38.4)	73 (18.2)		
Living with others^a^	202 (61.4)	127 (38.6)	329 (81.8)		
Ethnic groups				0.151	0.698
Han nationality	214 (61.1)	136 (38.9)	350 (87.5)		
Others	32 (64.0)	18 (36.0)	50 (12.5)		
Payment methods				21.144	<0.001
Self-pay	1 (50.0)	1 (50.0)	2 (0.5)		
Urban resident basic medical insurance	52 (64.2)	29 (35.8)	81 (20.1)		
Rural cooperative medical insurance	126 (72.0)	49 (28.0)	175 (43.5)		
Urban employee basic medical insurance	67 (47.5)	74 (52.5)	141 (35.1)		
Public medical care	1 (33.3)	2 (66.7)	3 (0.7)		
Monthly household income per capita, ¥^a^				30.150	<0.001
≤1,000	69 (76.7)	21 (23.3)	90 (22.4)		
1,001–3,000	123 (66.8)	61 (33.2)	184 (45.8)		
3,001–4999	46 (44.7)	57 (55.3)	103 (25.6)		
≥5000	9 (36.0)	16 (64.0)	25 (6.2)		
Occupation status				26.804	<0.001
Employed	19 (65.5)	10 (34.5)	29 (7.2)		
Unemployed	153 (72.5)	58 (27.5)	211 (52.5)		
Retired	75 (46.3)	87 (53.7)	162 (40.3)		
Residence				15.904	<0.001
Rural areas	78 (72.9)	29 (27.1)	107 (26.7)		
Town and county	80 (67.2)	39 (32.8)	119 (29.7)		
City	89 (50.9)	86 (49.1)	175 (43.6)		
Comorbidity				3.436	0.064
No	35 (51.5)	33 (48.5)	68 (16.9)		
Yes	212 (63.5)	122 (36.5)	334 (83.1)		
Total prescription medications per day				12.708	0.002
≤3	99 (52.4)	90 (47.6)	189 (47.0)		
4–5	96 (68.1)	45 (31.9)	141 (35.1)		
≥6	52 (72.7)	20 (27.8)	72 (17.9)		
Frequency of daily doses				3.478	0.324
1	110 (65.1)	59 (34.9)	169 (42.0)		
2	59 (61.5)	37 (38.5)	96 (23.9)		
3	75 (58.1)	54 (41.9)	129 (32.1)		
4	3 (37.5)	5 (62.5)	8 (2.0)		
Stroke subtype				0.188	0.664
Ischemic stroke	208 (61.0)	133 (39.0)	341 (84.8)		
Hemorrhagic stroke	39 (63.9)	22 (36.1)	61 (15.2)		
BMQ specific-necessity score	16.22 ± 3.16	17.86 ± 2.75	16.85 ± 3.11	−5.497	<0.001
BMQ specific-concerns score	14.97 ± 3.64	13.74 ± 3.91	14.50 ± 3.79	3.140	0.002
Health literacy score	59.11 ± 8.78	66.19 ± 9.53	61.84 ± 9.70	−7.477	<0.001

^a^ Living with others includes living with spouse, children, and nanny.

### Risk factors for medication non-adherence

All variables with a *p*-value < 0.05 in the univariate analysis were included in the logistic regression analysis. Table 4 shows the results of the multivariable analyses. Results from logistic regression suggest that educational level and total prescription medications per day as well as the BMQ specific-necessity, BMQ specific-concerns, and health literacy scores were associated with medication non-adherence.

**TABLE 4:** Factors associated with medication non-adherence using logistic regression.

Variable	B	S.E.	Wald χ^*2*^	*p*	OR (95%CI)
Educational level					
Primary school and below	-		13.646	0.003	–
Junior school	-0.821	0.291	7.971	0.005	0.440 (0.249, 0.778)
Senior Secondary and junior college	−1.002	0.305	10.823	0.001	0.367 (0.202, 0.667)
Bachelor degree or above	−1.039	0.494	4.425	0.035	0.354 (0.134, 0.932)
Total prescription medications per day					
≤3			9.679	0.008	
4∼5	0.690	0.263	6.861	0.009	1.993 (1.190, 3.339)
≥6	0.803	0.334	5.771	0.016	2.233 (1.159, 4.300)
Health literacy score	−0.064	0.014	20.006	<0.001	0.938 (0.913, 0.965)
BMQ specific-necessity score	−0.131	0.043	9.058	0.003	0.878 (0.806,0.955)
BMQ specific-concerns score	0.105	0.032	11.134	0.001	1.111 (1.044, 1.182)

B, B coefficient; SE, standard error; Wald, Wald chi-squared test; *p*, *p*-value; OR, odds ratio; CI, confidence interval. Significance taken at *p* < 0.05.

Compared to stroke survivors who had primary school or less educational levels, those with senior secondary and junior college educational levels [OR (95% CI) = 0.440(0.249, 0.778)] as well as those who had bachelor’s degree or above [OR (95% CI) = 0.367(0.202, 0.667)] had significantly decreased risks of medication non-adherence. Those with 4–5 types of total prescription medications per day had 1.993 times the risk of medication non-adherence as compared to those with ≤3 types [OR (95% CI) = 1.993(1.190, 3.339)]. On the other hand, those with ≥6 types of total prescription medications per day were 2.233 times likelier to be medication non-adherent as compared to those with ≤3 types [OR (95% CI) = 2.233(1.159, 4.300)]. Furthermore, when the BMQ specific-concerns score increased by one unit, the medication non-adherence increased by 11.1% [OR (95% CI) = 1.111(1.044, 1.182)]. Meanwhile, for every one-unit increase in the Health Literacy Scale for Stroke Patients score, there will be a 0.938 times decrease in the GMAS-C score (*β* = −0.641 (95% CI; (0.913, 0.965)). Lastly, for every one-unit increase in the BMQ specific-necessity score, there will be a 0.878 times decrease in the GMAS-C score (*β* = −0.131, (95% CI; 0.806, 0.995)).

## Discussion

To the best of our knowledge, this cross-sectional study is the first to evaluate medication non-adherence among older adult stroke survivors in China. Our findings showed that medication non-adherence was observed in 61.4% of the 402 sampled patients. Specifically, partial adherence, low adherence, and poor adherence were found to be 37.1%, 13.7%, and 10.7%, respectively. Moreover, the findings in terms of the non-adherence rates in the current study were much higher than those previously published in a meta-analysis of observational studies regarding post-stroke patients. The latter indicated that the overall non-adherence rate to secondary preventative medication among stroke survivors was 30.9%–35.9% (Al et al., 2016; Zhang et al., 2021). A possible explanation for this is that the aforementioned studies included all stroke patients aged over 18 years, while the present study focused on patients over 60 years of age. In this context, Yuvaraj et al. (2019) found that being part of the elderly age group is a determinant of non-adherence to medications after adjusting for possible confounding variables. Older adults are prone to multiple comorbidities and use more medications than their younger counterparts and may therefore present with a higher risk of medication non-adherence (Chiang-Hanisko et al., 2014). Taken together, our findings present concerns about the alarming rate of non-medication adherence among older adult stroke survivors in Chenzhou, Hunan Province, China, which requires further attention. This finding, while preliminary, suggests that strategies to promote medication adherence among this group are urgently needed.

Meanwhile, the educational level was found to be a predictor of medication non-adherence among older adult stroke survivors. According to our findings, patients who had higher educational levels were less likely to be non-adherent as compared to those who had attended only primary school or less. These results are consistent with those of other studies and suggest that higher education levels are associated with adherence (Kirkman et al., 2015; Jin et al., 2016; Bandi et al., 2017). This could be explained by the fact that those with higher educational levels may be less likely to have negative beliefs about medications (Lemay et al., 2018), which would promote medication adherence. Therefore, when seeking strategies to improve medication adherence among stroke survivors, educational-level factors must be considered. Meanwhile, it is worth noting that the average duration of education for stroke patients was significantly lower in patients aged ≥65 years than in patients aged <65 years (Lu et al., 2018). Such findings help shed light on the educational level as an important challenge for medication adherence interventions among older adult stroke survivors.

Among the common concerns, we found that the medication non-adherence of stroke survivors increased with that of total prescription medications per day. These results are congruent with the findings of earlier studies that indicate older adults are more adherent to a simplified medication regime (Jin et al., 2016). A possible explanation of this result is that patients may be less likely to forget to take medicine if the number of pills is low (Napolitano et al., 2016). However, Kim et al. (2020) reported that optimal medication adherence was associated with more prescribed medicines. This rather contradictory result could be attributed to the study including chronic diseases, while our study only considered elderly stroke patients.

Another important finding was that low needs or high concern regarding medication were associated with medication non-adherence. This finding is in agreement with previous studies on medication adherence among elderly people with chronic diseases (Lemay et al., 2018; Park et al., 2018), which revealed that as compared to patients with high needs and low concerns about medication, those with low needs and high concerns had significantly high medication non-adherence. Our results corroborated that of Horne and Weinman (1999b) who suggested that beliefs in medication may offer accurate predictability about adherence. Additionally, concerns about prescribed medications and unawareness of the rationale for treatments were expressed as primary reasons for non-adherence by stroke survivors (Bauler et al., 2014). Hence, for medical professionals, it is of great importance to outline and educate patients on the necessity of treatments rather than simply providing information about medication, while also managing patients’ concerns by sharing the known side effects of prescribed drugs and helping them recognize and cope with side effects to improve their confidence.

Overall, the most important clinically relevant finding was that health literacy is significantly associated with medication non-adherence. Here, health literacy refers to the degree to which individuals have the capacity to obtain, process, and understand basic health information and services needed to understand and use information to promote, maintain, or improve their health (Ratzan, 2001). According to this finding, lower health literacy has a greater likelihood of being associated with non-medication adherence. This result matches those observed in earlier studies (Lee et al., 2017; Mayo-Gamble and Mouton, 2018). This may be attributable to the fact that low health literacy is associated with less understanding of prescribed medication instructions (Mayo-Gamble and Mouton, 2018) and problems in using preventive services (Stormacq et al., 2019). As health literacy is considered the most important modifiable risk factor of socioeconomic differences in health (šedová et al., 2021), future work should investigate tailored interventions considering health literacy among older adult stroke survivors.

## Strengths and limitation

The most important strength of this study is that to the best of our knowledge, it provides invaluable information on the prevalence and factors associated with medication non-adherence by using standardized rating scales among older adult stroke survivors in Chenzhou, Hunan Province, China. Furthermore, our findings may provide a comprehensive picture of medication non-adherence among stroke survivors, which may lay the groundwork for interventions aimed at increasing adherence among this population. Here, it should be noted that our participant population was older adult stroke survivors, who have been frequently left out of studies. Hence, our study addresses an understudied group.

However, this study has limitations that are worth considering. First, information on medication adherence was collected for the previous months prior to the survey, so some degree of recall bias cannot be ruled out. This could lead to inaccurate estimations of the prevalence of medication non-adherence. Additionally, the data relied on self-reported practices of medication adherence, which might have been over- or under-reported by participants. Second, it was limited in scope. Participants were from three tertiary hospitals in Chenzhou, Hunan Province, China, which limits its generalizability to the broader regions in China. Third, this was a cross-sectional study. Therefore, associations between medication non-adherence and risk factors cannot necessarily be considered causal relationships.

## Conclusion

Our findings emphasize that medication adherence among older adult stroke survivors is problematic in this sample of study participants from Chenzhou, Hunan Province, China. Specifically, we found that medication non-adherence was significantly associated with educational levels, number of prescription medications to be taken per day, low needs or high concerns for medication, and health literacy. Building on this, we have offered specific recommendations for proposing population-specific medication adherence interventions among older adult stroke survivors. Our findings also suggest that efforts are needed to explore the prevalence and associated factors of medication non-adherence among older adult stroke survivors in other countries.

## Data Availability

The raw data supporting the conclusion of this article will be made available by the authors, without undue reservation.
